# Supplementary material for: NEO-STIM advances personalized neoantigen-specific adoptive T cell therapy
Source: Nat Commun. 2026 Feb 5;17:3683. doi: 10.1038/s41467-026-68680-1 (PMC13099984; doi:10.1038/s41467-026-68680-1)
Supplement: Supplementary file 1 — Supplementary Information [file 41467_2026_68680_MOESM1_ESM.pdf]

SUPPLEMENTARY INFORMATION:

SUPPLEMENTARY FIGURES

Supplementary Figure 1

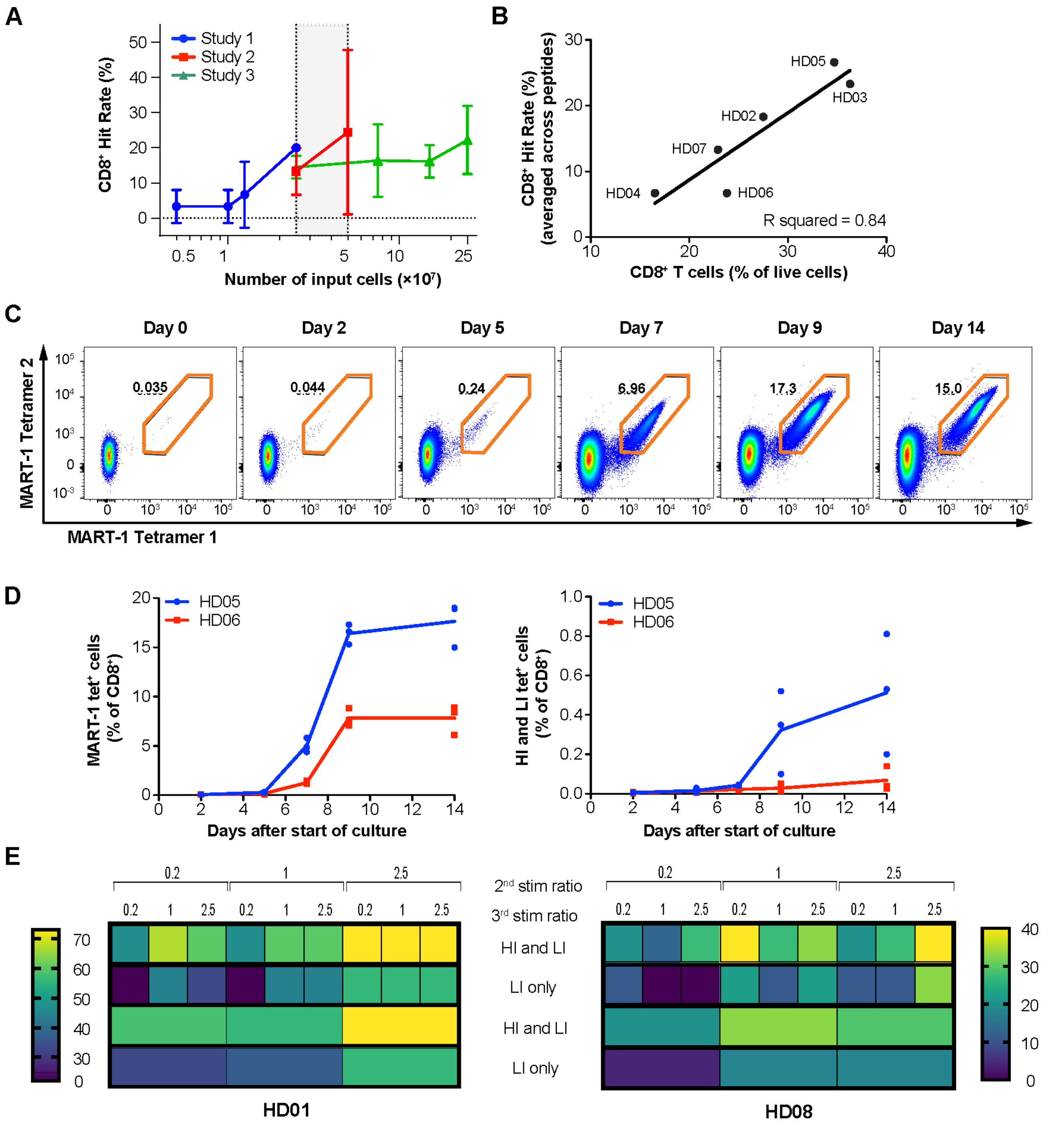

Supplementary Figure 1. **Critical parameters that influence CD8$^+$ priming, activation and expansion.**

A: Identification of cell input range. Impact of cell input number (post-CD14$^+$/CD25$^+$ depletion) on percent hit rate was studied in three sets of experiments with an overall testing range of 5 million to 250 million input cells per vessel. n=2 healthy donors (HDs) for study 1, n=3 HD for studies 2 and 3. Hit rate was defined as actual hits over number of possible hits among tested replicates. Data points are mean with SD. Grey shaded area is the cell input range chosen for manufacturing.

B: Correlation between frequency of CD8$^+$ T cells (as % of live cells) in starting material post-CD14$^+$/CD25$^+$ depletion and percent hit rate (day14) for high and low immunogenic peptides (see **Suppl Table 1**). This result supports the rationale of depleting cell populations which are either unnecessary or potentially inhibitory. Correlation was performed using simple linear regression method, R^2=0.84.

C-D: Growth kinetics of 1º culture.

C: Representative flow plots showing MART-1 tet$^+$ CD8$^+$ T cells (red polygon, as % of live CD8$^+$ cells) over different timepoints of 1º culture of NEO-STIM for HD05.

D: Kinetics of MART-1-specific CD8$^+$ T cells (left) and high and low immunogenic (HI and LI) antigen-specific CD8$^+$ T cells (right) in two healthy donors over different timepoints of 1º culture of NEO-STIM. n=3 replicates (independent cell samples from the same patient) with line graph connecting the mean percentage of tetramer$^+$ CD8$^+$ cells for each donor. While the highest percentage of antigen-specific T cells against MART-1 by and large was reached after 9 days, the responses against neoantigens increased further post day 9. The antigen-specific fraction was highest between days 9 and 14 after start of culture, consistent with prior reports[1]. Therefore, adding the 2º culture on day 14 of the 1º culture was adopted for the final protocol.

E: Optimization of the ratio of cell numbers from 2° and 3° culture to 1° and 2° culture. 1° cultures were restimulated with a 2° culture at ratios of 0.2, 1 and 5 (2° culture cells, post-depletion step, to 1° cells on day 14). In analogy, for the second restimulation the same ratios of cells were used. Experiment was set up with three biological replicates for each healthy donor. Since the hit rate for MART-1 was 100%, as expected (data not shown), here only hit rates without MART-1 are plotted, either all *de novo* neoantigen responses (HI and LI) or LI only. Heat maps represent percent hit rates for different ratios for two HDs. Upper two rows: all different ratios tested. Lower two rows: averages of the three ratios of the 3° culture. Looking at the two lower rows, the hit rates were typically higher at the 1 and 2.5 ratios compared to the 0.2 ratio, particularly for the responses against low immunogenic antigens. Looking at the impact of different ratios within the 3° cultures (two upper rows), the picture was more ambiguous. Note that in future iterations only one restimulation was used and therefore adding equal or greater numbers of new APCs and T cells at restimulation was adopted for the final protocol.

Source data are provided as a Source Data file.

Supplementary Figure 2

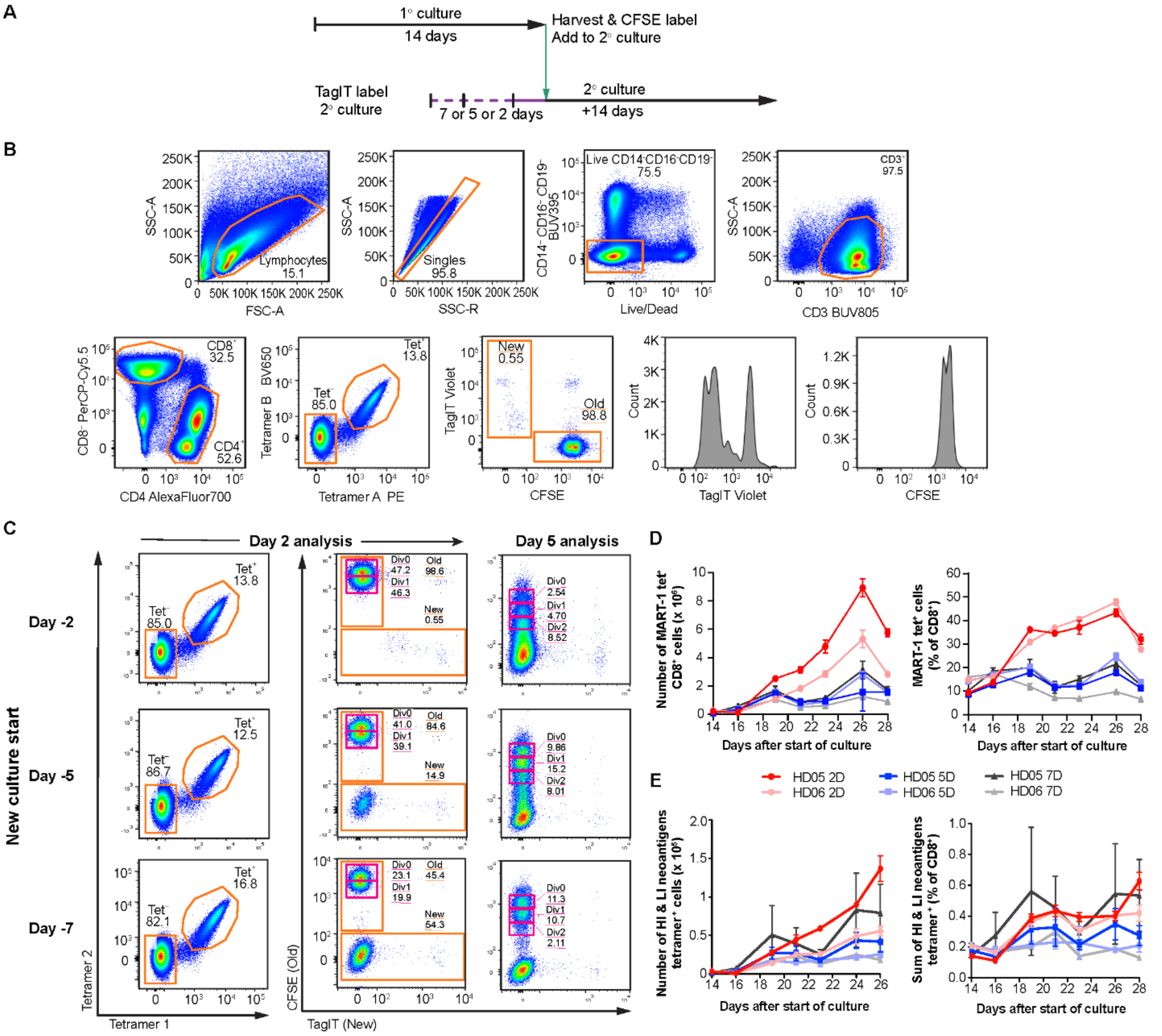

Supplementary Figure 2. Optimization of timing for 2⁰ culture initiation.

A. Schematic of 1⁰ cultures and 2⁰ cultures labeled with proliferation dyes to evaluate the priming and expansion of antigen-specific responses post-restimulation when the 2⁰ cultures are initiated two, five or seven days before restimulation.

B. Lineage gating flow cytometry analysis showing lymphocytes, single cells, live cells, $CD3^+$, $CD4^+$, $CD8^+$, $tetramer^+$ $CFSE^+$ and $tetramer^+$ $Tag\text{-}IT^+$ cell populations.

C. Flow cytometry plots show the percentage of tet$^+$ cells at day 2 after the start of combined culture (far left). The proliferation of 1° cultures (CFSE$^+$) and 2° cultures (Tag-IT$^+$) within the tetramer$^+$ CD8$^+$ T cell population from day 2 (middle panel) and day 5 (right panel) post-restimulation when 2° cultures were initiated at two, five or seven days before restimulation (top row, middle row and bottom row, respectively). With each T cell division, the intensity of the dye decreases, which allows for the assessment of proliferation on a per cell basis. Performing restimulation with cultures started 2 days prior resulted in robust proliferation of antigen-specific T cells from the 1° culture, as indicated by the significant dilution of CFSE, as well as some new priming events in the 2° culture at day 5 post-restimulation (tetramer+ Tag-IT+ T cells). On the contrary, performing restimulation with cultures that were prepared 7 days prior favored new priming events and minimal expansion of already primed cells, as illustrated by the larger fraction of tetramer$^+$ Tag-IT- cells and fewer cell divisions in the tetramer$^+$ CFSE$^+$ T cell population (potentially due to loss of APCs in the 2° culture by the day of restimulation). In summary, the time between preparing the 2° culture and the actual restimulation of the 1° culture impacts the balance of new priming events and expansion of already primed T cell responses.

D-E: Growth kinetics of 2° culture post-restimulation for responses against MART-1 (D) and high immunogenic (HI) and low immunogenic (LI) neoantigens (E). Absolute number of neoantigen-specific cells (left) and percentage of neoantigen-specific cells of all CD8$^+$ cells (right) at timepoints after start of culture (restimulation performed on day 14), when 2° cultures were initiated at two (red), five (blue), or seven (grey) days prior to restimulation. Data points represent mean and SD of three replicates. n=2 HD and n=3 technical replicates per condition. Note, that initiation of the 2° culture 2 days prior to restimulation led to the overall highest

percentages and highest absolute numbers of antigen-specific T cells. Additionally, the number of antigen-specific cells peaks at day 12 post 2nd stimulation in the majority of cases. Therefore, initiation of 2° culture two days prior to restimulation and harvest of combined culture after 12 days (total culture time 26 days) was chosen for future experiments.

Source data are provided as a Source Data file.

Supplementary Figure 3

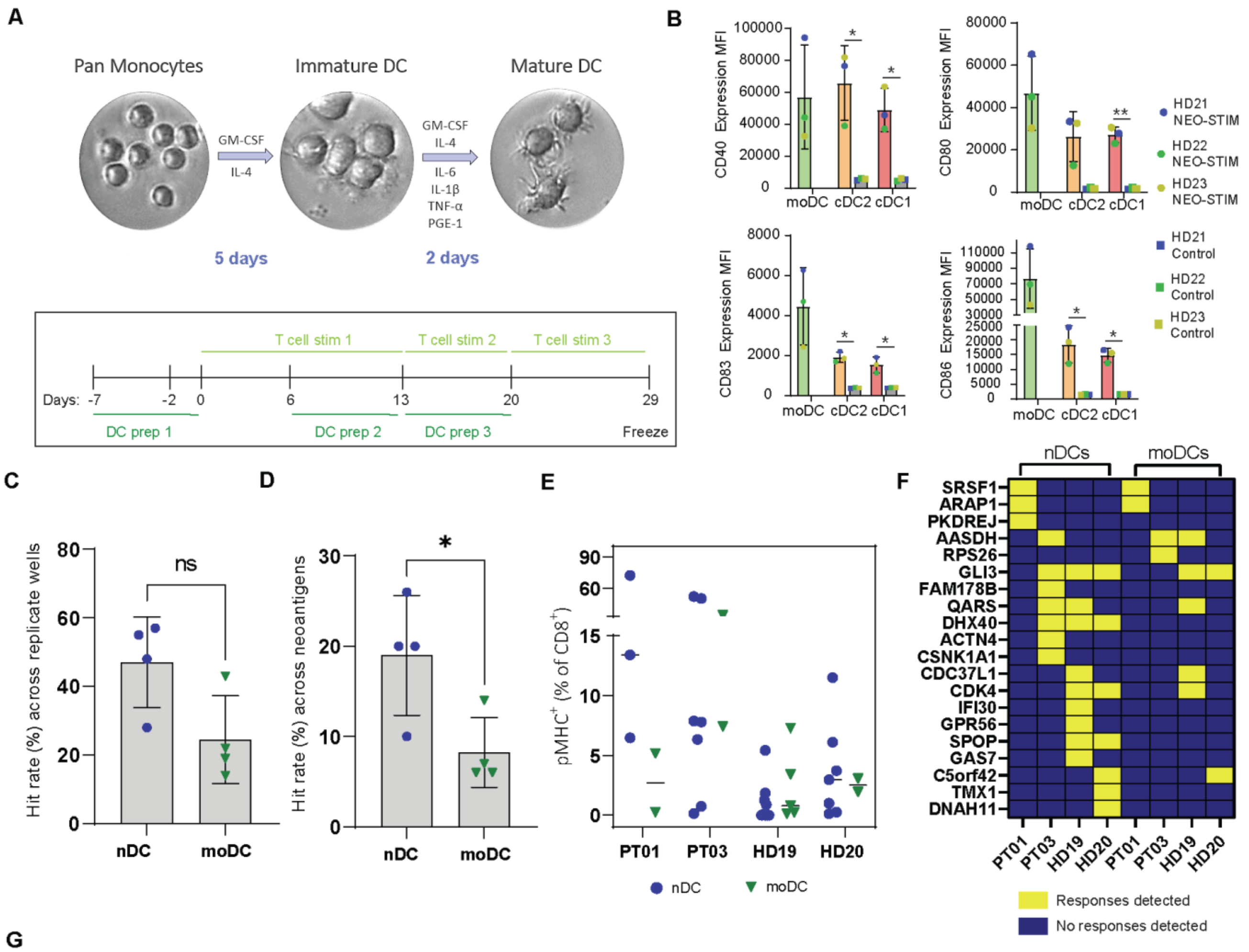

G

	nDC				moDC			
	CD14dep CD25dep				CD14dep CD25dep			
	PT01 (Melanoma patient)	PT03 (Melanoma patient)	HD19 (Healthy donor)	HD20 (Healthy donor)	PT01 (Melanoma patient)	PT03 (Melanoma patient)	HD19 (Healthy donor)	HD20 (Healthy donor)
Bulk Fold expansion (range)	83-582	48-849	30-219	12-61	14-94	176-2920	292-2890	30-210
Hit rate across replicate wells	28%	48%	57%	55%	22%	19%	43%	14%
Hit rate across neoantigens	3 out of 30	7 out of 35	9 out of 35	7 out of 35	2 out of 30	2 out of 35	5 out of 35	2 out of 35
Response to long peptide	Yes	Yes	NA	NA	No	No	NA	NA
Total absolute number x 10^6	838	933	18	31	5.3	362	254	9.5
Functionality	2/3	--	NA	NA	2/2	--	NA	NA

Supplementary Figure 3. Comparison of naturally circulating dendritic cells (nDCs) with monocyte-derived DCs (moDCs) as antigen presenting cells in NEO-STIM.

A. Workflow for preparing moDCs from $CD14^+$ monocytes to prime and expand antigen-specific T cells. Upper panel: Maturation of $CD14^+$ monocytes to mature monocyte-derived DCs (moDCs). Lower panel: Matured moDCs (DC prep 1, 2, 3) were added to CD14/CD25-depleted PBMCs at 1:10 ratio on days 0, 13 and 20 as indicated.

B. Expression levels of CD40, CD80, CD83 and CD86 were measured on mature monocyte-derived DCs, mature (left bar) and immature (right bar) cDC2 and cDC1 (n=3 HD per condition). Mean fluorescence intensity for each marker is reported. *P* values were defined with paired two-tailed t-test. *: $P \leq 0.05$, **: $P \leq 0.01$, exact *P* values can be found in the Source Data file.

C. – G. NEO-STIM using nDCs was compared with the analogous protocol using moDCs, measuring the outputs of hit rate across replicate wells (C), hit rate across neoantigens (D), frequency of neoantigen-specific tetramer $(tet)^+$ $CD8^+$ cells (E) and diversity of response (F). Hit rate across replicate wells was defined as number of replicate wells with responses over number of all replicates with possible responses across pools (PT01 – 6 pools; PT03, HD19 & HD20 – 7 pools). Hit rate was defined as actual hits over number of possible hits among tested replicates. Diversity in this context is defined as responses against multiple neoantigens. A summary of results for all parameters tested is shown (G). The protocol using nDCs was favorable in most conditions. For C & D data shown as mean with SD (n=4 donors, each datapoint shows average of technical triplicates) where *P* values were defined with unpaired t-test. ns, $P > 0.05$; *$P = 0.0311$. Horizontal lines in E represent median frequency.

Source data are provided as a Source Data file.

Supplementary Figure 4

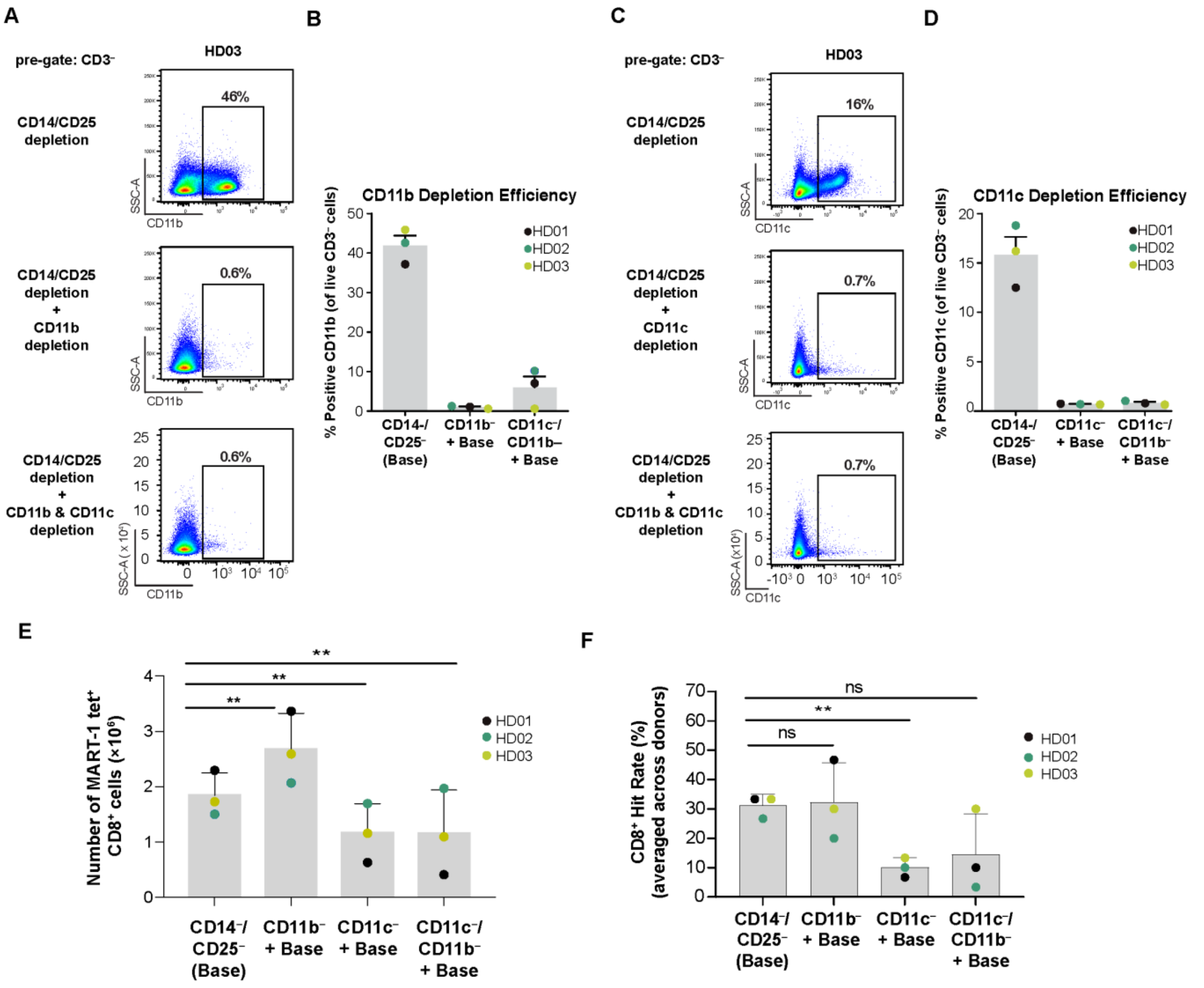

Supplementary Figure 4. Impact of CD11b$^+$ and CD11c$^+$ cell depletion on T cell responses.

A.-D.: Efficiency of CD11b$^+$ and CD11c$^+$ cell depletion.

A. Representative flow plots showing the frequency of CD11b$^+$ cells post-depletion of two cell subsets (CD14$^+$ and CD25$^+$ cells, base protocol), three cell subsets (CD14$^+$, CD25$^+$ and CD11b$^+$ cells) or four cell subsets (CD14$^+$, CD25$^+$, CD11b$^+$ and CD11c$^+$ cells). Data from donor HD03.
B. Bar graph showing frequency of CD11b$^+$ cells post-depletion of two cell subsets (CD14$^+$ and CD25$^+$ cells, base protocol), three cell subsets (CD14$^+$, CD25$^+$ and CD11b$^+$ cells) and four cell

subsets ($CD14^+$, $CD25^+$, $CD11b^+$ and $CD11c^+$ cells) across three healthy donors (HDs). Data shown as mean with SEM.

C. Representative flow plots showing frequency of $CD11c^+$ cells post-depletion of two cell subsets ($CD14^+$ and $CD25^+$ cells, base protocol), three cell subsets ($CD14^+$, $CD25^+$ and $CD11c^+$ cells) or four cell subsets ($CD14^+$, $CD25^+$, $CD11b^+$ and $CD11c^+$ cells) for HD03.

D. Bar graph showing frequency of $CD11c^+$ cells post-depletion of two cell subsets ($CD14^+$ and $CD25^+$ cells, base protocol), three cell subsets ($CD14^+$, $CD25^+$ and $CD11c^+$ cells) or four cell subsets ($CD14^+$, $CD25^+$, $CD11b^+$ and $CD11c^+$ cells) across three HDs. Data shown as mean with SEM.

E– F. Impact of $CD11b^+$ and $CD11c^+$ depletion on T cell response. A pool of peptides, comprised of one memory-like antigen (MART-1) and five model antigens for *de novo* responses (two highly immunogenic and three low immunogenic, see **Suppl Table 1**) was used for priming. Impact of depletion of APC subsets on number of antigen-specific responses for MART-1 (E) and on hit rate for model neoantigens (F) was evaluated. For each donor, hit rate was defined as actual hits over number of possible hits among n=6 technical replicates per donor. Data shown as mean with SDs of n=3 HD, where each datapoint is the average of 6 technical replicates of a donor. *P* values for E defined by multiple comparisons using FDR correction, for F by unpaired two-sided t-test. ns, $P > 0.05$, $^{**}P \leq 0.01$. tet^+: tetramer-positive, exact *P* values can be found in the Source Data file.

Source data are provided as a Source Data file.

Supplementary Figure 5

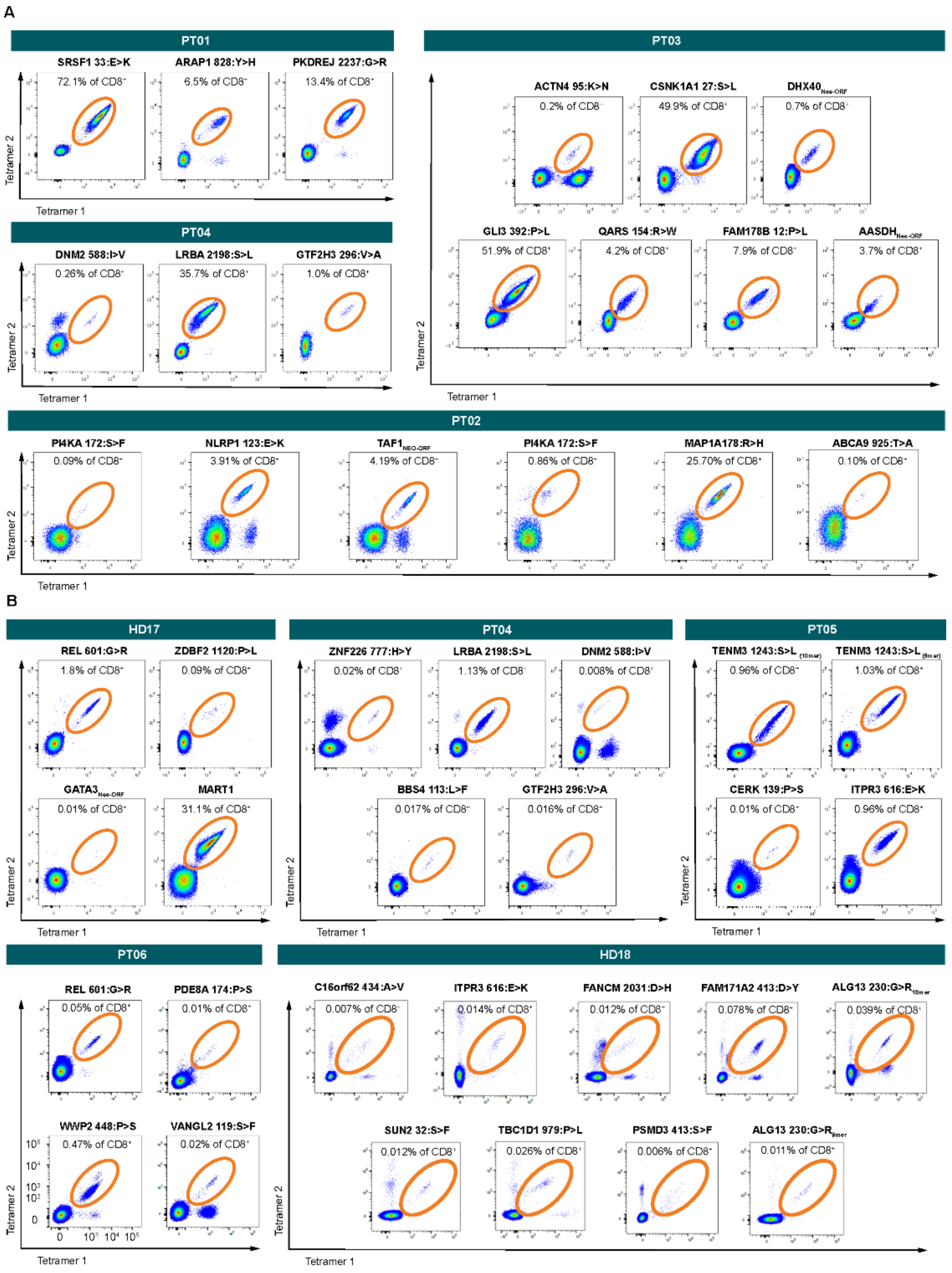

Supplementary Figure 5. Tetramer staining of neoantigen-specific T cells in drug product (DP) for healthy and patient (melanoma) donors.

Pseudocolor dot plots show tetramer (tet) staining of $CD8^+$ cells from research-scale DPs (A) or therapeutic-scale DPs (B). Tet^+ cells are depicted in orange circles and percentages are % tet^+ cells of all $CD8^+$ cells.

Supplementary Figure 6

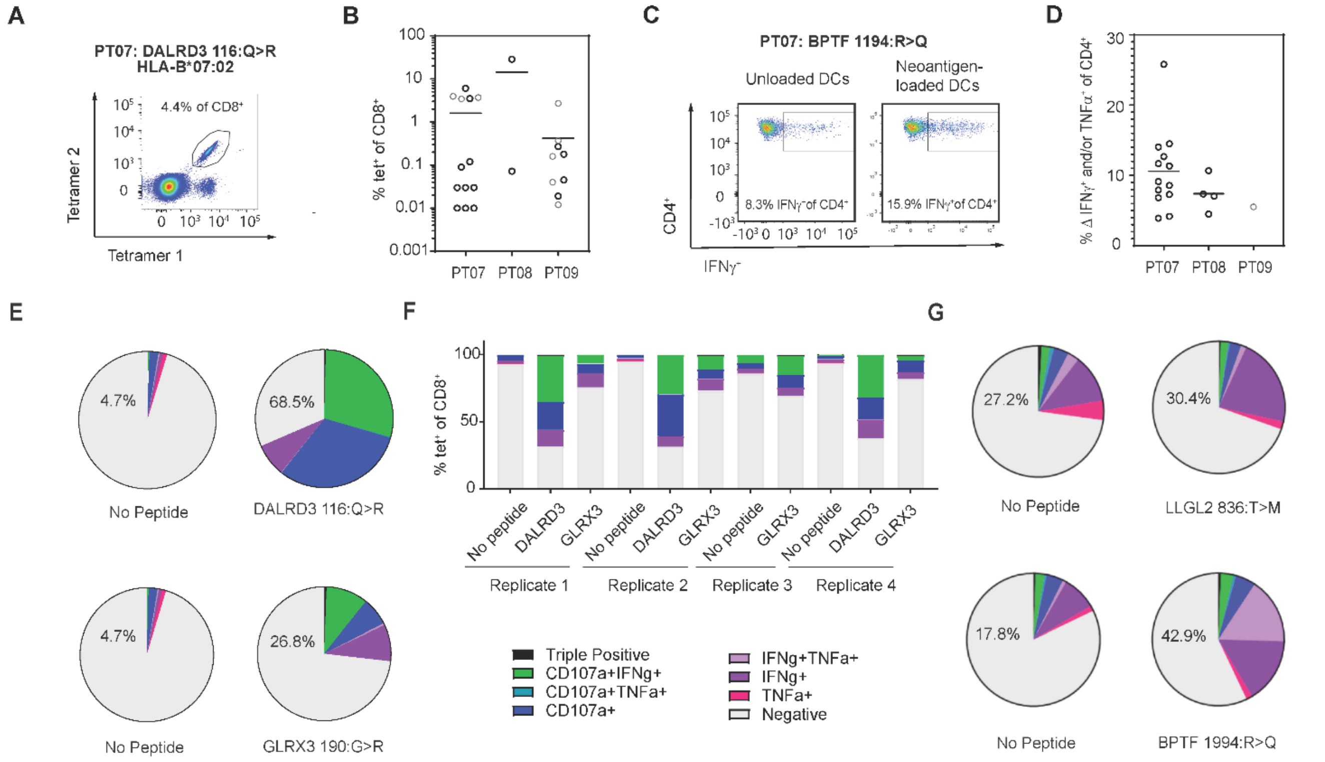

Supplementary Figure 6. NEO-STIM process applied to material from ovarian carcinoma patients at research scale.

NEO-STIM was applied to PBMCs derived from patients with ovarian cancer (PT07-09) using personalized tumor antigens and model antigens that were identified for each patient as immunogens. The optimized protocol (**Fig. 1B**) was used.

A. Example flow cytometry plot, showing percentage of tetramer^{+}(tet^{+}) CD8^{+} T cells at the end of NEO-STIM from patient PT07. Aggregated data in B.

B. Frequencies of neoantigen-specific CD8^{+} T cells across all three patients (in each case the highest frequency out of the four replicates). Data points represent frequency of an induced response post-NEO-STIM. Light grey data points represent responses to model antigens. Horizontal lines show mean frequency.

C. Example flow cytometry plot showing frequency of IFNγ^{+} cells for induced CD4^{+} cells co-cultured with mutant (MT) neoantigen-loaded dendritic cells (DCs) versus unloaded DCs in post-NEO-STIM samples for a response of PT07. Aggregated data in D.

D. Frequency of neoantigen-specific CD4^{+} T cells across all patient samples. Data points represent the delta of IFNγ and/or TNFα (as a percent of CD4^{+} T cells) when rechallenged with MT neoantigen-loaded DCs versus unloaded DCs in post-NEO-STIM samples (in each case the highest frequency out of the four replicates). Light grey data points represent responses to model antigens. Horizontal lines show mean frequency.

E. – G. Only responses with sufficient sample availability and magnitude of response were tested.

E. Representative examples of two responses from well 2, PT07. Pie charts depict the polyfunctionality of the identified neoantigen-specific CD8^{+} T cells (gated on tet^{+} CD8^{+} cells) upon rechallenge with MT neoantigen-loaded DCs versus unloaded DCs. Percent numbers above pie charts indicate the percentage of functional cells with one, two or three functions.

F. Stacked bar charts depicting polyfunctionality of all measured replicate wells (n = 4) of the identified neoantigen-specific CD8^{+} T cell responses (n = 2) from PT07. Percent numbers on y axis represent frequency of the response as measured within this assay.

G. Pie charts depict the polyfunctionality of the identified neoantigen-specific CD4^{+} T cells (gated on IFNγ^{+} and/or TNFα^{+} CD4^{+} cells) upon rechallenge with MT neoantigen-loaded DCs versus unloaded DCs (PT07). Percent numbers above pie charts indicate the percentage of functional cells with one, two or three functions. Averages of n=3 technical replicates shown.

Source data are provided as a Source Data file.

Supplementary Figure 7

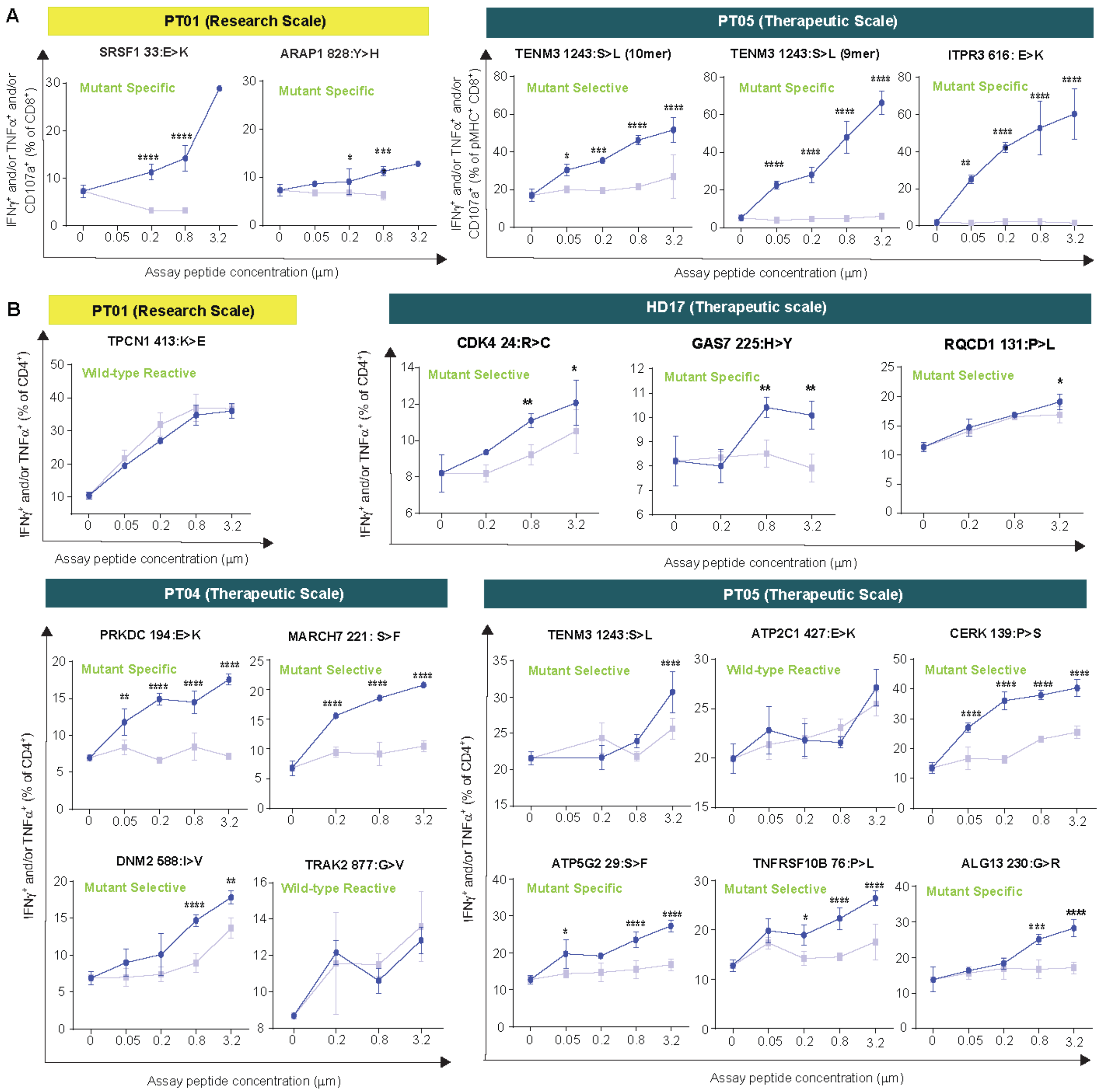

Supplementary Figure 7. Induced T cell responses are mutant-reactive.

Data for all experiments from runs at research scale (yellow headers) or therapeutic scale (dark green headers) for $CD8^+$ (A) and $CD4^+$ (B) responses. Drug products were co-cultured overnight with

unloaded dendritic cells (DCs) and DCs loaded with mutant (MT) or wild-type (WT) assay peptide at a range of concentrations (0.05 – 3.2 μM). Upregulation of IFNγ, TNFα, and/or CD107a was measured using flow cytometry. Each plot represents an experiment for a given mutation. $SRSF1_{33:E>K}$, $ARAP1_{828:Y>H}$ and $TPCN1_{413:K>E}$ responses were from experiments at research scale, some of which are also shown in **Fig. 2G**. The other responses are from the manufacturing process at therapeutic scale, some of which are also shown in **Fig. 4D**, see text for specificity definitions. Data shown as mean with SD (*n*=3 technical replicates) where *P* values of MT over WT were defined with Tukey's multiple comparison test; except SRSF1 33:E>K, ARAP1 828:Y>H and TPCN1 413:K>E (research scale): Data shown as mean with SD (*n*=1-3 technical replicates) where *P* values of MT over WT were defined using paired t-test with FDR correction for adjusted *P* value; and ITPR3 616:E>K: Data shown as mean with SD (*n*=1-3 technical replicates) where *P* values of MT over WT were defined with Sidak's multiple comparison test. *P ≤ 0.05; **P ≤ 0.01; ***P ≤ 0.001; ****P ≤ 0.0001, exact *P* values can be found in the Source Data file.

Source data are provided as a Source Data file.

Supplementary Figure 8

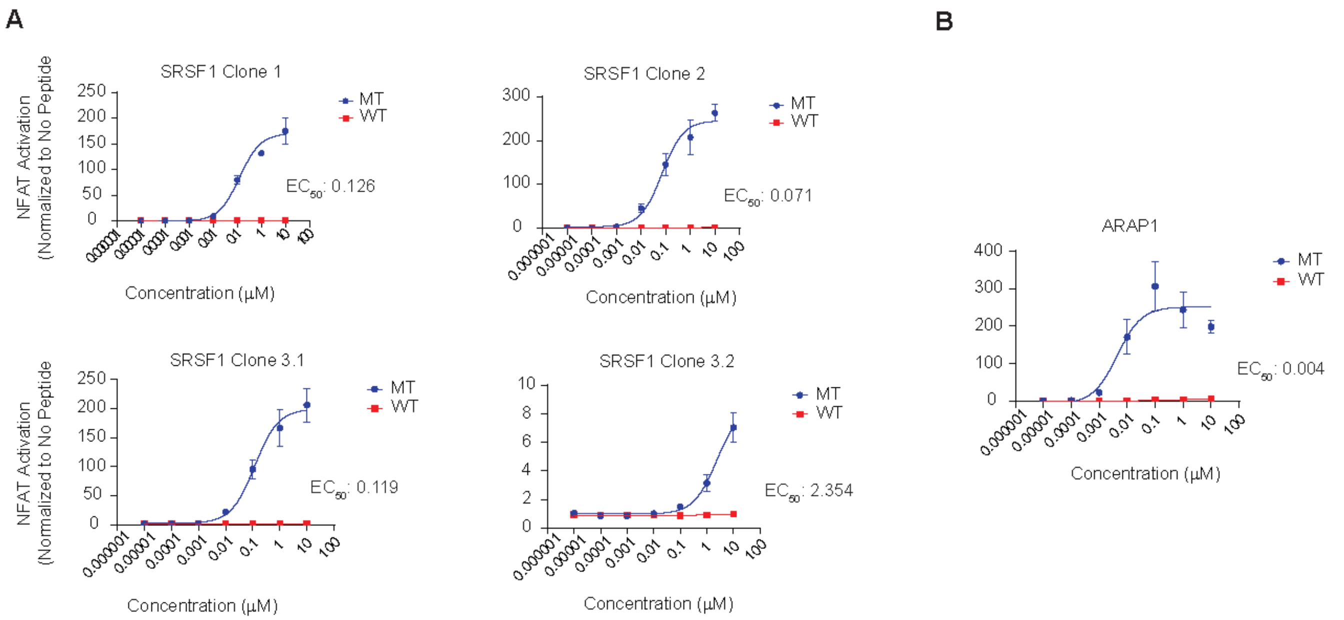

Supplementary Figure 8. Avidity measurements.

Neoantigen-specific VDJ regions from TCRs of the DP were sequenced, alpha and beta chain RNA generated, and transfected into NFAT Jurkat cells to measure functional avidity. EC_{50}: Peptide concentration resulting in half maximal effect. SRSF1 clone 3 was associated with two distinct TCR clonotypes, each defined by unique α/β chain pairings. Data points show mean (NFAT activation) and standard deviation (n=4, technical replicates).

Source data are provided as a Source Data file.

Supplementary Figure 9

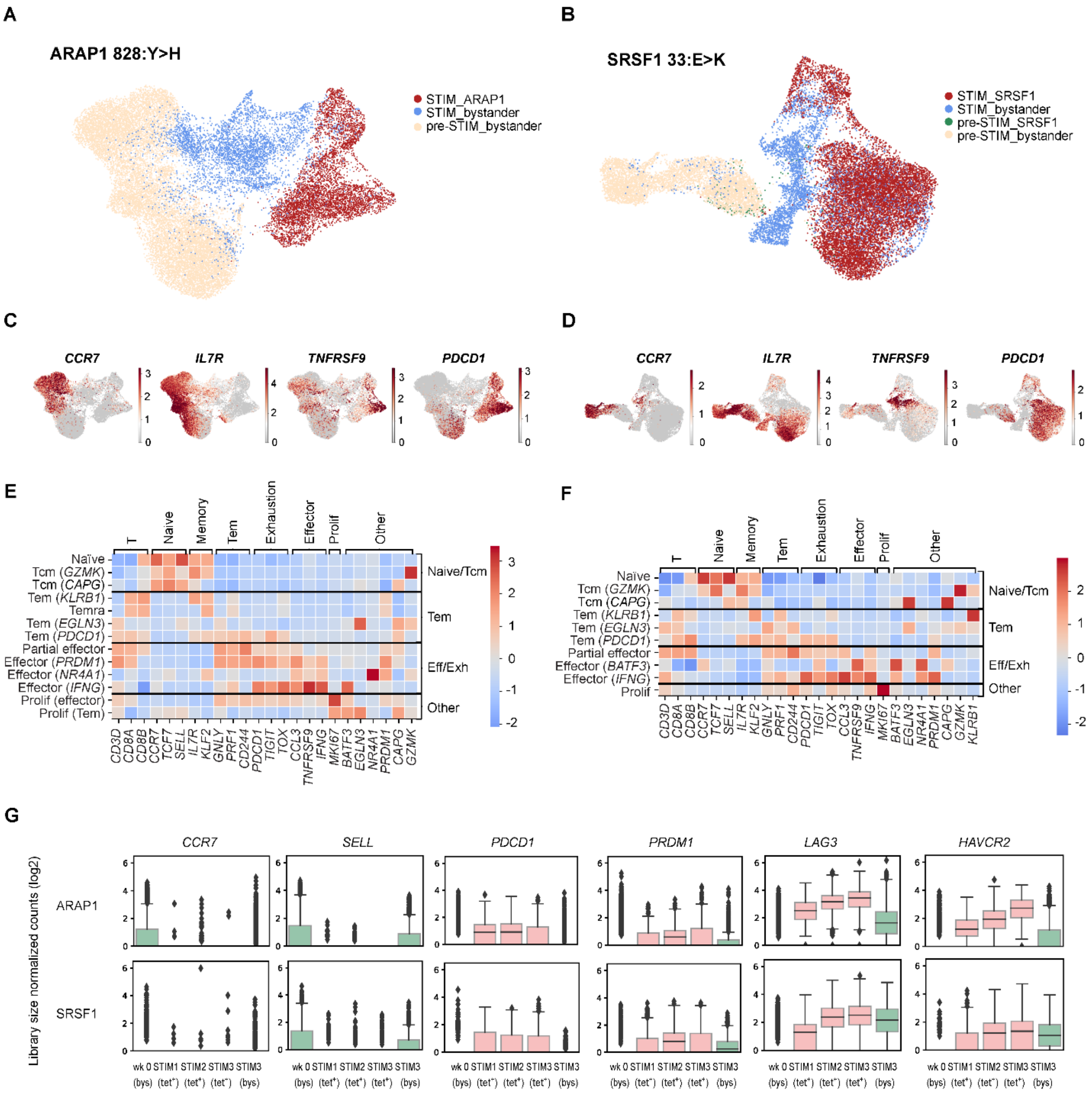

Supplementary Figure 9. Expression signatures of ARAP1$_{828:Y>H}$ and SRSF1$_{33:E>K}$ responses.

A. B. Uniform manifold approximation and projection (UMAP) of multimodal single-cell data of two T cell responses, ARAP1$_{828:Y>H}$ (A) and SRSF1$_{33:E>K}$ (B), showing clustering of antigen-

specific T cells in starting material (pre-STIM) and post-NEO-STIM (STIM) samples for neoantigen-specific and bystander cells.

C. D. Expression of representative marker genes for $ARAP1_{828:Y>H}$ (C) and $SRSF1_{131:E>K}$ (D) responses. T cells were observed from naïve (*CCR7*), memory (*IL7R*), effector (*TNFRSF9*), and exhausted (*PDCD1*) populations.

E. F. Heatmap showing expression of marker genes used to assign T cell phenotypes of $ARAP1_{828:Y>H}$ (E) and $SRSF1_{33:E>K}$ (F) responses. Left Y axis: Assigned phenotypes; right Y axis: Broader phenotype groupings. Lower X axis: marker genes; upper X axis: marker genes grouped by function. Expression values were Z-score-normalized within each gene.

G. Box plots showing expression of stem-like (*CCR7, SELL*) and exhaustion markers (*LAG3, PDCD1, PRDM1*) for ARAP1 (top) and SRSF1 (bottom). Expression profiles of bystander cells (bys) at the start (wk 0) and the end of the process (wk 3) were compared with the expression profiles of antigen-specific T cells (tet^+) at the end of stimulation 1, 2 and 3 (STIM1, STIM2 and STIM3, respectively). Expression values were log2 counts normalized for library size. Box plots show median and data quartiles. Outliers (whiskers) were defined using 1.5 times the interquartile range.

Source data are provided as a Source Data file.

Supplementary Figure 10

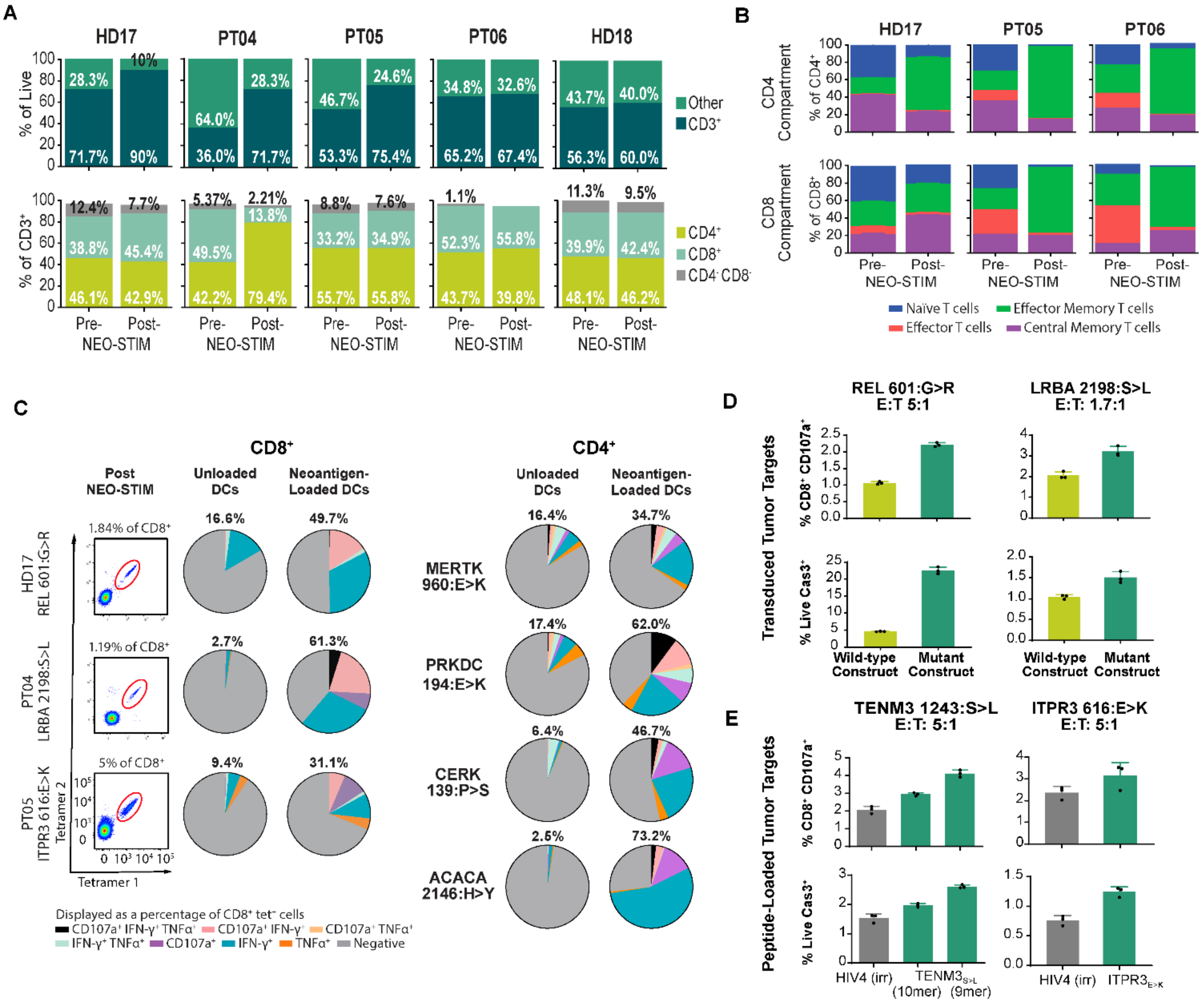

Supplementary Figure 10. Additional characterization of therapeutic-scale drug products (DPs).

A: Characterization of DP cell types by flow cytometry: Top row shows $CD3^+$ frequency as a fraction of live cells. Bottom row shows $CD8^+$ and $CD4^+$ T cells as a fraction of live $CD3^+$ T cells. n = 5 drug products.

B: T cell differentiation status for $CD4^+$ cells (top) and $CD8^+$ cells (bottom). Central memory T cells (T_{cm}): $CD62L^+$ $CD45RA^-$, Effector Memory T cells (T_{em}): $CD62L^-$ $CD45RA^-$, Effector T cells (T_{eff}):

CD62L$^-$ CD45RA$^+$, naïve T cells ($T_{naïve}$): CD62L$^+$ CD45RA$^+$. Representative examples from eight phenotypic panels performed across the study (research scale and therapeutic scale, single measurements for 3 drug products).

C: Example flow cytometry plots with tetramer$^+$ fraction of CD8$^+$ T cells indicated on the left. Representative polyfunctionality pie charts from 26 responses tested, showing tetramer$^+$ CD8$^+$ T cells (middle) and CD4$^+$ T cells (right), with or without re-challenge with mutant neoantigen-loaded DCs, n = 1-3 technical replicates. All 26 responses tested showed to be polyfunctional.

D, E: Cytotoxicity of T cell responses post NEO-STIM from HD17 and PT04 samples. Mobilization of CD107a on CD8$^+$ T cells (top panels) and active caspase 3 on tumor cells (bottom panels) was measured upon co-culture. Six responses tested and found cytotoxic, of which five are shown here. Data shown as mean with SD; n=3 technical replicates. D: Responses against untransduced tumor cells (parental A375 line with patient-specific HLA allele) or tumor cells transduced with a 200 amino acid long mutant or wild-type neoantigen-containing construct and the patient-specific HLA allele. E: Responses against unloaded or cognate peptide loaded tumor cells transduced with patient-specific HLA allele.

Source data are provided as a Source Data file.

Supplementary Figure 11

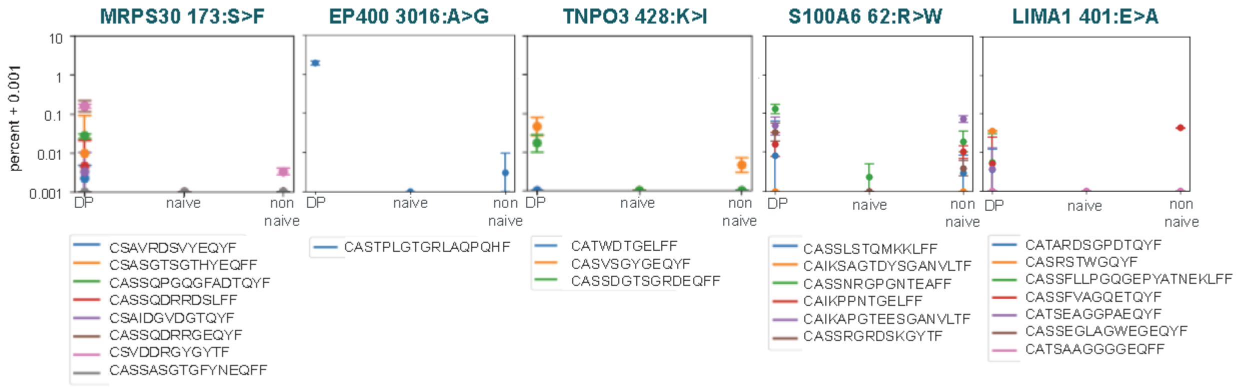

Supplementary Figure 11. Bulk TCR sequencing NAC01.

Tracking of five different neoantigen-specific responses in the DP and blood of patient NAC01[45] by bulk TCR sequencing at the clonotype level. The blood was separated into naïve and non-naïve fractions by bead-based separation: pan-T cells were isolated to positively select non-naïve cells, leaving naïve cells in the flow through (Miltenyi Biotec kits 130-096-535; 130-097-095). The error bar shows the individual data for two replicates with the data points representing the average (frequency of clonotypes).

Source data are provided as a Source Data file.

SUPPLEMENTARY TABLES:

Supplementary Table 1: Model antigens used in process development

Sequence	Gene Name	Classification	Immunogenicity	protein change
ELAGIGILTV	MART-1	Memory-like antigen[23] (main reference list)	N/A	N/A
MLTGPPARV	GATA3	Neoantigen discovered and validated at BioNTech US (NEON)	High	395: +1 Frameshift
ALNSEALSV	TMPRSS2: ERG	Neoantigen discovered and validated at BioNTech US (NEON)	High	Fusion
FIASNGVKLV	ACTN4	Neoantigen described in the literature[24] (main reference list	Low	95:K>N
GLFGDIYLAI	CSNK1A	Neoantigen described in the literature[22] (main reference list	Low	27:S>L
SLYNTVATL	HIV3	Viral antigen[2]	Low	Non-mutated

Supplementary Table 2: Demographics of donors used for development of NEO-STIM process

	PT01	PT02	PT03	PT04	PT05	PT06	PT07	PT08	PT09
**Age**	55-60	55-60	35-40	55-60	65-70	65-70	75-80	60-65	65-70
**Tumor type**	Melanoma	Melanoma	Melanoma	Melanoma	Melanoma	Melanoma	Ovarian CA	Ovarian CA	Ovarian CA
**Medication at the time of leukapheresis**	Cortico-steroid	No Cortico-steroid	No Cortico-steroid	Cortico-steroid	No Cortico-steroid	No Cortico-steroid	No Cortico-steroid	No Cortico-steroid	No Cortico-steroid
**Pathology results**									
**Detected driver mutation**	ARID1A, BRAF, CDKN2A	BRAF V600E	N/A	BRAF V600E	BRAF, NRAS & cKIT WT	NRAS	TP53 mutation (primary)	WT-1, PAX8, p53 positive	FOXL2 mutation
**Inclusion criteria/stage of disease**									
**Performance status (ECOG)**	≤1	≤1	≤1	≤1	≤1	≤1	≤1	≤1	≤1
**Disease stage**	Stage IV	Stage IV	Stage IV	Stage IV	Stage IV	Stage IV	≤Stage III	≤ Stage III	≤ Stage III
**Previous treatments**									
**1st line**	Check-point inhibitor	Other Therapy	Check-point inhibitor	Check-point inhibitor	Check-point inhibitor	Check-point inhibitor	Other Therapy	N/A	N/A
**2nd line**	N/A	N/A	N/A	Other Therapy	N/A	N/A	N/A	N/A	N/A
**3rd line**	N/A	N/A	N/A	Check-point inhibitor	N/A	N/A	N/A	N/A	N/A
**Leukocytes (10^9/L)[1]**	3.0-6.0	3.0-6.0	> 6.0	>6.0	>6.0	3.0-6.0	3.0-6.0	>6.0	.0-6.0

[1]on day of apheresis, prior to apheresis; Abbreviations: PT: Patient; M: male; F: female; N/A – not applicable; WT - wild-type; ECOG: Eastern Cooperative Oncology Group; PD-1: programmed cell death protein 1; BRAF/MEKi: Inhibitor of BRAF and MEK.

REFERENCES (ONLY IN SUPPLEMENT)

1 Lissina A, B. O., Afonso G, Larsen M, Gostick E, Price DA, Mallone R, Appay V. Priming of Qualitatively Superior Human Effector CD8+ T Cells Using TLR8 Ligand Combined with FLT3 Ligand - PubMed. *Journal of immunology (Baltimore, Md. : 1950)* **196** (2016). https://doi.org/10.4049/jimmunol.1501140

2 Kan-Mitchell, J. *et al.* The HIV-1 HLA-A2-SLYNTVATL Is a Help-Independent CTL Epitope. *The Journal of Immunology* **172**, 5249-5261 (2004). https://doi.org/10.4049/jimmunol.172.9.5249
