# Supplementary material for: NEO-STIM advances personalized neoantigen-specific adoptive T cell therapy
Source: Nat Commun. 2026 Feb 5;17:3683. doi: 10.1038/s41467-026-68680-1 (PMC13099984; doi:10.1038/s41467-026-68680-1)
Supplement: Supplementary file 2 — Description of Additional Supplementary Files [file 41467_2026_68680_MOESM2_ESM.pdf]

Description of Additional Supplementary Files

Supplementary Data 1: List of T cell responses and expression data

Supplementary Data 2: List of flow antibody panels and fluorochromes
