# Supplementary material for: NEO-STIM advances personalized neoantigen-specific adoptive T cell therapy
Source: Nat Commun. 2026 Feb 5;17:3683. doi: 10.1038/s41467-026-68680-1 (PMC13099984; doi:10.1038/s41467-026-68680-1)
Supplement: Supplementary file 5 — Reporting Summary [file 41467_2026_68680_MOESM5_ESM.pdf]

nature portfolio

Corresponding author(s): Marit van Buuren

Last updated by author(s): 2026/01/12

# Reporting Summary

Nature Portfolio wishes to improve the reproducibility of the work that we publish. This form provides structure for consistency and transparency in reporting. For further information on Nature Portfolio policies, see our Editorial Policies and the Editorial Policy Checklist.

## Statistics

For all statistical analyses, confirm that the following items are present in the figure legend, table legend, main text, or Methods section.

| n/a | Confirmed | |
|---|---|---|
| [ ] | [x] | The exact sample size (*n*) for each experimental group/condition, given as a discrete number and unit of measurement |
| [ ] | [x] | A statement on whether measurements were taken from distinct samples or whether the same sample was measured repeatedly |
| [ ] | [x] | The statistical test(s) used AND whether they are one- or two-sided<br>*Only common tests should be described solely by name; describe more complex techniques in the Methods section.* |
| [x] | [ ] | A description of all covariates tested |
| [x] | [ ] | A description of any assumptions or corrections, such as tests of normality and adjustment for multiple comparisons |
| [ ] | [x] | A full description of the statistical parameters including central tendency (e.g. means) or other basic estimates (e.g. regression coefficient) AND variation (e.g. standard deviation) or associated estimates of uncertainty (e.g. confidence intervals) |
| [ ] | [x] | For null hypothesis testing, the test statistic (e.g. *F*, *t*, *r*) with confidence intervals, effect sizes, degrees of freedom and *P* value noted<br>*Give P values as exact values whenever suitable.* |
| [x] | [ ] | For Bayesian analysis, information on the choice of priors and Markov chain Monte Carlo settings |
| [x] | [ ] | For hierarchical and complex designs, identification of the appropriate level for tests and full reporting of outcomes |
| [x] | [ ] | Estimates of effect sizes (e.g. Cohen's *d*, Pearson's *r*), indicating how they were calculated |

*Our web collection on statistics for biologists contains articles on many of the points above.*

## Software and code

Policy information about availability of computer code

| | |
|---|---|
| Data collection | No code was used for data collection. |
| Data analysis | Alignment and mutational calling, and neoantigen peptide selection: proprietary custom bioinformatics analysis; flow cytometry: FlowJo, v07-10; Statistical analyses: Python language (version 3.9), or GraphPad Prism (version 7.04-9.5.1).TCR repertoires: MiXCR 4.3.2 |

For manuscripts utilizing custom algorithms or software that are central to the research but not yet described in published literature, software must be made available to editors and reviewers. We strongly encourage code deposition in a community repository (e.g. GitHub). See the Nature Portfolio guidelines for submitting code & software for further information.

## Data

Policy information about availability of data

All manuscripts must include a data availability statement. This statement should provide the following information, where applicable:

- Accession codes, unique identifiers, or web links for publicly available datasets
- A description of any restrictions on data availability
- For clinical datasets or third party data, please ensure that the statement adheres to our policy

Source data for the data described in this manuscript are provided. DNA and RNA sequencing data are not publicly available for contractual obligations. Based on ethical approval: the data can be made available upon request for future research purposes involving adoptive cell therapies for the treatment of advanced cancers; it however cannot be used for publications outside of this study. All data provided are anonymized to respect the privacy of patients who have participated in line with applicable laws and regulations. Data requests pertaining to the manuscript may be made to the corresponding author (M.M.v.B.,

marit.vanbuuren@biontech.us). Requests will be processed within 16 weeks. The remaining data are available within the Article, Supplementary Information or Source Data file.

## Research involving human participants, their data, or biological material

Policy information about studies with human participants or human data. See also policy information about sex, gender (identity/presentation), and sexual orientation and race, ethnicity and racism.

| | |
|---|---|
| Reporting on sex and gender | Sex was ascertained at screening by the investigator in the respective studies. Given the study size, outcomes were not analyzed per sex. Sex is not reported to protect privacy of the participants. |
| Reporting on race, ethnicity, or other socially relevant groupings | Socially constructed or socially relevant categories were not used in this manuscript. |
| Population characteristics | Information on age, sex, ECOG performance status, cancer type, site and stage, detected driver mutation, leukocyte count and pretreatments were collected for participants in clinical trials from which leukapheresis materials were used, some of which are reported in Supplementary Table 2 in a form which protects data privacy of the participants. Of the above parameters, number of pretreatment cancer regimens, ECOG performance status, detected driver mutation, leukocyte count, age, and sex are potential covariates. No formal subanalyses based on population characteristics were conducted. |
| Recruitment | Healthy Donor Material was sourced commercially. Patient material was from clinical studies in which participants were recruited by the investigators / clinical trial centers per eligibility criteria outlined in the study protocols. Prior to any non-routine analysis, sampling or intervention, each patient was fully informed about the study, and signed an informed consent form. Patients were not compensated for participating in the study. This manuscript does not discuss clinical study outcomes per primary and secondary endpoints, so bias resulting from recruitment is not relevant in this context. |
| Ethics oversight | Clinical studies from which patient material was derived were approved by the Institutional Review Board at each participating site. |

Note that full information on the approval of the study protocol must also be provided in the manuscript.

# Field-specific reporting

Please select the one below that is the best fit for your research. If you are not sure, read the appropriate sections before making your selection.

[x] Life sciences    [ ] Behavioural & social sciences    [ ] Ecological, evolutionary & environmental sciences

For a reference copy of the document with all sections, see nature.com/documents/nr-reporting-summary-flat.pdf

# Life sciences study design

All studies must disclose on these points even when the disclosure is negative.

| | |
|---|---|
| Sample size | Sample size determinations for initial method development were based on ethical and cost considerations, since human samples were required (Bacchetti 2005; doi: 10.1093/aje/kwi014). Feasibility and value of information gained was also taken into consideration, given that experimental setups were complex and new information gained was considered highly valuable. A method for ex vivo generation of T cell responses against predetermined personalized tumor antigens did not exist, as such, we chose sample sizes based on our prior experience in method development. Exploratory method optimization experiments: 2-4 individual donors coupled with replication of experiments and inclusion of biological replicates (typically 3) for the first exploratory optimizations were considered to generate meaningful results. Protocol validation experiments: Two sequential experimental series at two scales (research and therapeutic scale) with 3-5 individual donors each (total of 9 patients) and 1-4 biological replicates in each experiment were considered sufficient to corroborate method optimization results. Sample sizes for post-infusion studies requiring specific clinical samples were based on availability; volume and number of samples was limited because of the fact that most of the patient material had already been used for translational research within the clinical study (Borgers et al.). |
| Data exclusions | Data was excluded as reported and justified in Figure captions of Figure 4A, B. |
| Replication | Samples were replicated three or four times where applicable, and data is represented as mean and standard deviation, unless noted differently in Figure caption. Detailed replication data is found in Supplementary Data Set 1. |
| Randomization | There were no clinical or animal groups to allocate samples to in this in vitro study. |
| Blinding | Blinding was not applicable in this study. |

# Reporting for specific materials, systems and methods

We require information from authors about some types of materials, experimental systems and methods used in many studies. Here, indicate whether each material, system or method listed is relevant to your study. If you are not sure if a list item applies to your research, read the appropriate section before selecting a response.

## Materials & experimental systems

| n/a | Involved in the study | |
|---|---|---|
| ☐ | ☒ | Antibodies |
| ☐ | ☒ | Eukaryotic cell lines |
| ☒ | ☐ | Palaeontology and archaeology |
| ☒ | ☐ | Animals and other organisms |
| ☒ | ☐ | Clinical data |
| ☒ | ☐ | Dual use research of concern |
| ☒ | ☐ | Plants |

## Methods

| n/a | Involved in the study | |
|---|---|---|
| ☒ | ☐ | ChIP-seq |
| ☐ | ☒ | Flow cytometry |
| ☒ | ☐ | MRI-based neuroimaging |

# Antibodies

| | |
|---|---|
| Antibodies used | A list of all antibodies is provided in Supplemental Dataset S2. |
| Validation | Antibodies were validated by Flow cytometry by the manufacturers, as indicated in Supplemental Dataset S2. |

# Eukaryotic cell lines

Policy information about cell lines and Sex and Gender in Research

| | |
|---|---|
| Cell line source(s) | A375 cells and Jurkat cells were from ATCC (CRL-1619; TIB-152). Further sub cell lines were created in house. |
| Authentication | Cell line authentication was performed by the manufacturer via STM profiling. No further authentication has been performed. |
| Mycoplasma contamination | Not all cell lines / sub cell lines were tested for mycoplasma contamination. When tested, they were negative for mycoplasma contamination. |
| Commonly misidentified lines (See ICLAC register) | A375 and Jurkat cell lines are not listed on the ICLAC register of misidentified cell lines (version 13), nor in the list of cross-contaminated cell lines (Capes-Davis et al. Int J Cancer 127:108, 2010). |

# Plants

| | |
|---|---|
| Seed stocks | *Report on the source of all seed stocks or other plant material used. If applicable, state the seed stock centre and catalogue number. If plant specimens were collected from the field, describe the collection location, date and sampling procedures.* |
| Novel plant genotypes | *Describe the methods by which all novel plant genotypes were produced. This includes those generated by transgenic approaches, gene editing, chemical/radiation-based mutagenesis and hybridization. For transgenic lines, describe the transformation method, the number of independent lines analyzed and the generation upon which experiments were performed. For gene-edited lines, describe the editor used, the endogenous sequence targeted for editing, the targeting guide RNA sequence (if applicable) and how the editor was applied.* |
| Authentication | *Describe any authentication procedures for each seed stock used or novel genotype generated. Describe any experiments used to assess the effect of a mutation and, where applicable, how potential secondary effects (e.g. second site T-DNA insertions, mosiacism, off-target gene editing) were examined.* |

# Flow Cytometry

## Plots

Confirm that:

☐ The axis labels state the marker and fluorochrome used (e.g. CD4-FITC).

☒ The axis scales are clearly visible. Include numbers along axes only for bottom left plot of group (a 'group' is an analysis of identical markers).

☒ All plots are contour plots with outliers or pseudocolor plots.

☒ A numerical value for number of cells or percentage (with statistics) is provided.

## Methodology

| | |
|---|---|
| Sample preparation | Blood draws or leukapheresis were performed to collect PBMCs from the patients or healthy donors. PBMCs were isolated through Ficoll or washing steps. PBMCs or manufactured Drug Product were frozen in DMSO-containing media. Materials were thawed and used in downstream assays. |
| Instrument | Flow cytometry instruments used were BD LSR Fortessa for analysis and BD FACSAria was used for sorting. |

| | |
|---|---|
| Software | For flow cytometry analysis the software FlowJo, versions 07 - 10 were used. |
| Cell population abundance | Single cell sorting was performed on BD FACSAria and purity of sorted populations was promoted by setting conservative gates for lineage markers/pMHC multimers and utilizing the "purity sort mode" to restrict the presence of cells in adjacent droplets during sorting. Approximate processed cell number for each different condition was as follows:<br>- PBMC CD8+ bulk: ~6,000 cells<br>- DP CD8+ pMHC- bystanders: ~2,000 cells<br>- DP CD8+ pMHC+: ~50-2,000 cells Purity of samples post-sort was evaluated post library generation by cell type categorization utilizing transcriptome and targeted proteome profiles, and final analysis only included CD8+ cells.<br>- TCRbeta libraries were prepared from snap-frozen T cell pellets derived from PBMC or tumor digest: 1 million pan T cells each |
| Gating strategy | Gating strategies for the different experiments are described in the Online Methods. An example for gating strategy is shown in Supplementary Figure 2B. |

☒ Tick this box to confirm that a figure exemplifying the gating strategy is provided in the Supplementary Information.
